# Governance Considerations for Point-of-Care Ultrasound: a HIMSS-SIIM Enterprise Imaging Community Whitepaper in Collaboration with AIUM

**DOI:** 10.1007/s10278-024-01365-7

**Published:** 2025-01-03

**Authors:** Irene W. Y. Ma, Michael L. Francavilla, Jason T. Nomura, Adam Kielski, Francisco Fernandez, Kevin Piro, Rachel Liu, Josephine Valenzuela, Michael Toland, Jessica Koehler, Gregg Cohen, Monief Eid, Wilson Choi, James D. Nolan, Robinson M. Ferre, Morgan P. McBee, Tobias Kummer, Michael J. Lanspa, Jenn Quattrone Brown, Kristen DeStigter, Stella Desyatnikova, Allan Bottemiller

**Affiliations:** 1 Department of Medicine, University of Calgary, 3330 Hospital Dr. NW, Calgary, AB T2N 4N1 Canada; 2 Department of Radiology, Whiddon College of Medicine, University of South Alabama, Mobile, AL USA; 3 Department of Emergency Medicine, Christiana Care Health System, Newark, DE USA; 4 Amazon Web Services, Senior Partner Solutions Architect, Seattle, WA USA; 5 Department of Emergency Medicine, College of Medicine, University of Cincinnati, Cincinnati, OH USA; 6 Division of Hospital Medicine, Division of General Internal Medicine, Oregon Health & Science University, Portland, OR USA; 7 Department of Emergency Medicine, Yale School of Medicine, New Haven, CT USA; 8 Valleywise Health, University of Arizona College of Medicine, Phoenix, AZ USA; 9 Creighton University School of Medicine, Phoenix, AZ USA; 10 University of Maryland Medical System, Baltimore, MD USA; 11 Department of Emergency Medicine, University of Michigan Medical School, Ann Arbor, MI USA; 12 Emergency Medicine, Veterans Affairs Ann Arbor Healthcare System, Ann Arbor, MI USA; 13 Radiology and Imaging Sciences, National Institutes of Health Clinical Center, Bethesda, MD USA; 14 E-Health Portfolio, Ministry of Health, Riyadh, Kingdom of Saudi Arabia; 15 Enterprise Information Technology Services, NYC Health and Hospitals Corporation, New York, NY USA; 16 Information Technology, Valley Medical Center, Renton, WA USA; 17 Department of Emergency Medicine, Indiana University School of Medicine, Indianapolis, IN USA; 18 Department of Radiology and Radiological Science, Medical University of South Carolina, Charleston, SC USA; 19 Department of Emergency Medicine, Mayo Clinic, Rochester, MN USA; 20 Intermountain Critical Care Echocardiography Service, Intermountain Medical Center, Salt Lake City, UT USA; 21 Independent Consultant, Garland, TX USA; 22 Department of Radiology, University of Vermont Larner College of Medicine, Burlington, VT USA; 23 The Stella Center for Facial Plastic Surgery, Seattle, WA USA; 24 Independent Consultant, Kirkland, WA USA

**Keywords:** Point-of-care ultrasound, Enterprise imaging, Governance, Archive, Imaging informatics, System integration

## Abstract

Point-of-care ultrasound (POCUS) has emerged as a standard of care across a variety of healthcare settings due to its ability to provide critical clinical information and as well as procedural guidance to clinicians directly at the bedside. Implementation of enterprise imaging (EI) strategies is needed such that POCUS images can be appropriately captured, indexed, managed, stored, distributed, viewed, and analyzed. Because of its unique workflow and educational requirements, reliance on traditional order-based workflow solutions may be insufficient. To improve patient care outcomes and operational efficiency, a robust governance committee for POCUS within healthcare systems that addresses pertinent institutional policies to ensure effective and sustainable implementation of enterprise imaging, appropriate to the specific clinical encounter-based workflow needs of POCUS, is critical. This white paper explores several key governance considerations in the formulation and structure of a POCUS enterprise imaging strategy, focusing on program governance, clinical governance, technology governance, information governance, and financial governance.

## Introduction

Point-of-care ultrasound (POCUS) is performed directly at the bedside by the treating clinician, such as physicians, nurses, and advanced practice providers, to answer focused clinical questions, facilitate management decisions, and guide bedside procedures [1]. The use of POCUS for resuscitation, diagnosis, clinical management, and procedural guidance is associated with high diagnostic accuracy [2–4], no radiation exposure [5], and lower procedural complication rates than when no POCUS was used [6]. As a result, POCUS use is now considered the standard of care in some clinical settings and recommended by a number of organizations [7–11]. Failing to use it when indicated is a potential medicolegal risk [12, 13].

Enterprise imaging is a set of strategies to optimize the capturing, indexing, management, storage, distribution, viewing, exchange, and analysis of medical images including POCUS [14]. As the number of POCUS users and POCUS devices proliferate across healthcare systems in multiple disciplines and specialties, the importance of establishing and deploying a cohesive enterprise imaging strategy for POCUS became evident. Benefits to the archival of images that are viewable and discoverable include facilitating collaborative care, minimizing unnecessary repeat imaging, and fulfilling billing and legal requirements in some jurisdictions [14]. Additionally, the need to archive images and document POCUS findings is in alignment with best practice standards set forth by a number of clinical societies and organizations [8, 15–17].

Imaging departments traditionally employ an order-based image capture and storage process, whereby an order and request to perform imaging are first placed in the electronic health record (EHR) [18]. Two key differences in POCUS workflows make it challenging to adopt the traditional order-based imaging workflow. First, POCUS examinations are often unsolicited. The treating clinician performs POCUS, and the type(s) and number of anatomic areas imaged vary and are often not pre-determined. For example, for a patient presenting with hypotension and delirium, a clinician may intend initially to evaluate the heart, lungs, and inferior vena cava. However, upon noticing a red swollen knee at the bedside, the clinician may then proceed to evaluate for the presence of a knee effusion with POCUS. In such an example, it is challenging to rely solely on order-based imaging processes. Therefore, imaging solutions that allow for encounter-based imaging workflow (EBIW) processes are critical, whereby imaging performed is unsolicited [18]. However, not all POCUS practices are identical. While the need for EBIW is ubiquitous in departments such as critical care and emergency medicine, other settings such as specialized dermatology clinics, gastroeneterology clinics, or obstetrics clinics, where both patient flow and anatomic areas imaged may be more predictable, an order-based workflow solution may be preferred. The heterogeneous nature of POCUS practices across specialties and settings requires flexible solutions. Enterprise imaging solutions should ideally be able to support both workflows. A second key difference in the practice of POCUS compared to traditional imaging department workflows that make the implementation of enterprise imaging solutions more complex is the common need for ongoing training and quality assurance (QA) processes in POCUS. While sonography in imaging departments is performed by (or at least closely supervised by) already trained and credentialed sonographers, in the vast majority of clinical departments across specialties that perform POCUS, due to the training demands of many residency programs, both credentialed and non-credentialed users perform POCUS exams. In addition, these POCUS exams may be done for clinical reasons (clinical exams) and/or educational reasons (educational exams). Educational exams are commonly acquired solely for the purposes of training and, therefore, may have no implications on clinical care. As such, depending on local requirements, educational exams may not require reporting or archival in the EHR. Furthermore, an educational exam may become a clinical exam if clinically relevant findings are discovered during QA image review. For example, if an educational cardiac scan on a patient reveals the presence of a vegetation, this finding has direct implications for subsequent patient investigation and management decisions, including ordering confirmatory consultative imaging studies. Therefore, POCUS image management processes for documentation and report generation, assignments for documentation signoffs, and archival processes need to take this unique training aspect of POCUS into account. Given the observed and expected growth of POCUS use across specialties, implementing facility-wide enterprise imaging strategies requires both an understanding of and careful consideration of its complexity.

This white paper is the first of a number of white papers seeking to outline best practices for implementing enterprise imaging strategies for POCUS practices, which, in some cases, may include extending enterprise imaging strategies to include POCUS. This paper is the result of a unique collaboration between the Healthcare Information and Management Systems Society (HIMSS), the Society for Imaging Informatics in Medicine (SIIM), and the American Institute of Ultrasound in Medicine (AIUM). This work began in July 2023 when members of one of the three organizations with clinical and/or technical expertise in POCUS imaging and workflows formed the HIMSS-SIIM/AIUM POCUS workgroup. This group met monthly to discuss strategies to enhance POCUS workflows by establishing best practices. This present paper will discuss the importance of a robust governance and decision-making committee(s), as well as institutional policies to oversee the enterprise imaging strategy for POCUS within healthcare systems. It will focus on the importance of the following recommended governance framework for a POCUS program that includes program governance, clinical governance, technology governance, information governance, and financial governance. Whether these governance groups are five discrete entities or a single governing body that addresses these five key functions will be at the discretion of each healthcare system. Regardless of the governance setup, clear transparent communication is critical in effecting change.

## Program Governance

The overall purpose of POCUS program governance is to build and implement a sustainable POCUS enterprise imaging strategy and ensure the overall success of the POCUS program. Program governance creates a structure for coordinating decisions regarding POCUS, aligns the POCUS strategy with the overall enterprise imaging strategy, provides accountability for the program, facilitates resource allocation, and ensures that all stakeholders’ interests are considered [19].

### Goals

**Fig. 1:**
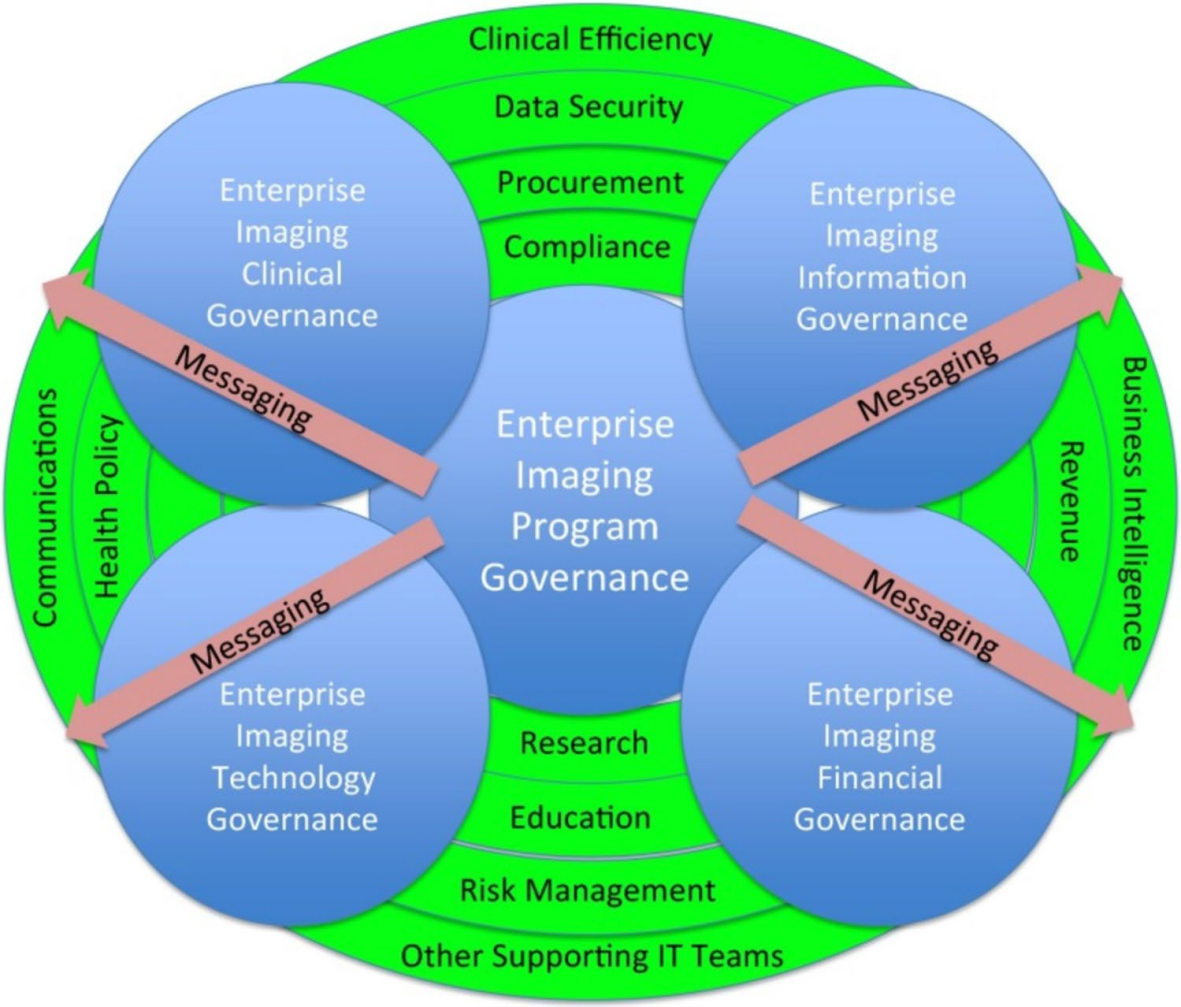
Enterprise imaging program governance and its integration with other governance areas. From Roth CJ, Lannum LM, Joseph CL. Enterprise imaging governance: HIMSS-SIIM Collaborative white paper. J Digit Imaging 2016;29:539–546

Specific goals of the POCUS program governance include but are not limited to establishing priorities for implementation, overseeing training and educational programs, documentation and archival processes, policies and standards, billing compliance, infection control policy, continuous quality assurance, and standards for credentialing and privileging (Fig. 1) [20]. The program should establish key metrics of success that ideally are specific, measurable, achievable, relevant, and time-bound. In some centers, program governance would also oversee POCUS research needs. Specific to policies and training recommendations, for specialties, institutions, or regions that have developed their own training recommendations and policies, these should be followed where relevant [21–23]. In these cases, program governance can simply help support or endorse these recommendations. For specialties without specific recommendations, program governance can offer guidance on key aspects of needed recommendations. The program governance should also define standard processes for image and clinical documentation in conjunction with EHR governance to have a uniform and standard location of information in the medical record to promote visibility and communication for patient care. Additionally, documentation and image retention should support appropriate billing, as billing compliance is a key driver for sustainability. Promoting audit readiness and maintaining clinical user trust are important. Coordination with relevant groups to ensure that institutional policies on infection control, information integrity, data security, and regulatory compliance policies are followed is also within the scope of program governance. Additionally, given the central role of the POCUS program governance, opportunities exist in goal alignment and coordination of resource-sharing between different groups of POCUS users. For example, there may be opportunities for improving educational efficiency, such as shared education and didactic teaching resources, simulation labs and a pool of standardized patients, quality improvement or research projects, and POCUS case reviews. Program governance should also be prepared to make strategic decisions to allow governance bodies to compromise when competing demands are present.

### Culture and Makeup

The POCUS program governance may consist of a single large, diverse group or a smaller executive group that delegates specific functions to other departmental groups [24]. Regardless of its makeup, program governance *must* have institutional support and leadership imprimatur. Leadership will provide overall strategic direction to the group and endorse and support the utilization of scarce resources, such as the time of information technology (IT) staff and biomedical engineers. Being able to align the POCUS strategy with the larger enterprise strategy of the institution is also important as it allows the POCUS program to help further the goals of the institution. For example, if the enterprise seeks to improve access to imaging-guided procedures to decrease the length of stay, program governance can assist with leveraging and coordinating necessary resources to support the creation of an appropriately trained procedure team.

Successful governance implementation requires that all necessary stakeholders be involved in the decision-making process from the beginning. Stakeholders from several areas will need to come together including clinical departments or specialties, IT, imaging/picture archiving and communication systems (PACS), biomedical engineering, research departments, quality improvement/assurance, billing, and equipment procurement. Identifying the clinical stakeholders may be challenging in large systems without already well-established POCUS workflows. Including representation from each area and department allows stakeholders to be aware of the growth needs of the organization and have a voice at the table where the broad scope of POCUS program imaging strategy deployment will occur over time. In this way, the program governance also serves as an executive forum for relationship building and knowledge sharing between imaging service lines and leaders. Failure to involve key departments and areas may lead to otherwise avoidable challenges down the line. For example, report templates created without the necessary guidance from billing compliance or equipment procurement may result in financial losses and reporting that is incompatible with the enterprise imaging storage workflow.

### Responsibilities

It is critical to define the boundaries of the POCUS program governance [19]. Some governance groups will govern all aspects of POCUS use, while others may focus on addressing imminent operational or billing/compliance needs. The landscape of POCUS is varied and imprecise and is not entirely based on devices, locations, providers, or the types of examinations performed [25]. Given that departmental practices, use cases, needs, and skills are heterogenous, defining where departments or program governance have jurisdiction may be challenging. Therefore, defining what constitutes POCUS and group responsibilities is an important beginning [24, 25]. Certain responsibilities may be delegated to other groups. For example, the program governance group will likely wish to delegate the authority to determine privileging criteria of POCUS practitioners to hospitals, departments, or clinical groups. In other cases, the program governance group may have minimal responsibilities. For example, the creation or implementation of a POCUS service line may be a multidisciplinary endeavor, and therefore, the creation of the components of the POCUS workflow may not fall upon the POCUS program governance. However, even in such cases, the program governance group can serve to assist with coordination among groups and provide accountability.

## Clinical Governance

As an increasingly diverse number of specialties are now using POCUS^1^, it is evident that the patient population and clinical conditions being evaluated by POCUS as well as POCUS workflows differ significantly among specialties. Therefore, there is no one-size-fits-all solution to POCUS enterprise imaging processes. The American Medical Association has affirmed that ultrasound privileges and training criteria be in accordance with recommendations and standards developed by each physician’s specialty (Policy H-230.960) [26]. Therefore, for meaningful clinical governance to occur, a variety of clinical stakeholders will need to be involved with the non-clinical and technological support teams in order to appropriately represent the breadth of POCUS use within an enterprise program.

### Goals

With this enterprise approach to POCUS, one goal that the different clinical specialties, environments, and stakeholders should move toward, as much as possible, is the standardization of their infrastructure, documentation, and image archival and retention processes. This standardization enables easier implementation, training, and familiarity by users across different locations and environments. Additionally, standardization of documentation and image archival processes maximizes compliance with the system’s standards for integration of POCUS information and images into the EHR to improve information availability and access. An order-based workflow decreases required manual data entry and improves downstream image storage and documentation for select POCUS practices where patient flow and anatomic areas imaged may be more predictable. For many other POCUS practices, such as in an emergency department, EBIW is more efficient and less prone to errors. In EBIW where the imaging event is unscheduled, the act of completing the POCUS examination process generates the imaging order retroactively [18]. The varied nature of POCUS practices is such that each specialty and/or physician group will need to determine its preferred workflow. The clinical governance group can then determine how to leverage the enterprise infrastructure to accommodate their desired workflow.

POCUS providers should be appropriately credentialed and privileged for the use of POCUS, according to the standards of their respective disciplines, specialties, and institutions, with consideration of state regulations and institutional policies [26]. Clinical privileging procedures and scope of practice for healthcare providers are defined by applicable regulatory agencies; a well-known example is the Joint Commission with its requirements for focused professional practice evaluation (FPPE) and ongoing professional practice evaluation (OPPE) for assessments of competency and quality [27]. In collaboration with the program governance, clinical governance should oversee QA processes, monitor ultrasound equipment and POCUS use, maintain standards for quality image acquisition and interpretation, and identify opportunities for improvements in POCUS performance and clinical applications [8, 28–30]. Individual POCUS programs may also seek accreditation to improve standardization of the processes and quality of the POCUS program. However, care should be taken to match the accreditation program to the clinical POCUS program within the enterprise to align quality and performance measures.

### Culture and Makeup

As the program expands, there will be initiation of POCUS use in new specialties and/or expansion of POCUS use into novel practice settings. Expansion of the POCUS program should continue to adhere to the enterprise standards that the program governance leadership has developed. It is important to have clinical stakeholders in these new areas identify champions serving to educate on workflow and standards along with providing support and mentorship for their colleagues. These champions are also able to inform adjustments to the enterprise solutions to meet the diverse needs of users and scenarios as expansion occurs.

### Responsibilities

Education, training, and acquisition of proficiency should occur in relation to specialty-specific guidelines to meet privileging requirements [31]. For specialties newly adopting the use of POCUS and do not have a clinical champion for the education and training of new providers, the clinical governance leadership can assist in identifying and/or providing expertise to help with training and educating the local leaders and champions [32]. These individuals will eventually be able to continue with the training of their colleagues and serve as key stakeholders in the continued enterprise approach to clinical governance. These clinical stakeholders are also key in the quality management process for both the enterprise and local programs.

With the additional clinical expansion of POCUS, the need for ultrasound hardware and devices to expand in numbers and system/vendor variety is inevitable. With continuing advancement in the miniaturization of hardware, novel transducer construction, artificial intelligence integration, image processing, and workflow integrations, it is necessary for a clinical governance approach that includes stakeholder evaluation regarding the clinical need for novel technology and the ability to integrate into the enterprise system with consideration of available resources.

In summary, effective clinical governance should include the following:

- A variety of relevant clinical stakeholders
- Standardization of documentation and image archival processes
- Determining preferences and needs of physician groups for order-based workflow vs. EBIW
- Leveraging the enterprise infrastructure to accommodate preferred workflow solutions
- Overseeing (or delegating) credentialing and privilege processes, QA processes, monitoring of ultrasound equipment and POCUS use, maintaining standards for quality image acquisition and interpretation, and identifying quality improvement opportunities
- Providing support and mentorship to specialties newly adopting POCUS
- Evaluating equipment and new technology

## Technology Governance

### Goals

Technology governance is a foundational element crucial for the seamless operation and optimization of a POCUS program. It serves as the backbone for overseeing the technical, management, and interoperability aspects of the entire POCUS infrastructure. Within this framework, one key area of focus is the effective management of ultrasound device connectivity and integration. This involves ensuring the seamless integration of POCUS devices with the existing healthcare information systems such as the EHR, picture archiving and communication system (PACS), vendor neutral archive (VNA), and reporting systems to facilitate viewing and reporting of images and image archival, as well as ensuring appropriate billing and coding practices and ultimately improving clinical decision-making.

### Culture and Makeup

How POCUS integration and technology governance will look will differ based on the capability and capacity of each hospital and/or healthcare organization. Smaller or less complex organizations may need time to develop the necessary processes. Since POCUS is part of standard medical care for many specialties with differing needs, we recommend that hospital and healthcare technology leaders actively partner with clinicians to address security, device acquisition and maintenance, and workflow needs. A realistic long-term plan that is co-developed by both clinical and information technology experts is critical to the success of any POCUS program. The importance of alignment of POCUS workflows with the broader clinical objectives, regulatory frameworks, and any relevant technical limitations within the healthcare system cannot be overstated. This alignment ensures that the use of ultrasound technology is not only safe and effective but also supports the overall goals of patient care and quality improvement initiatives within the healthcare system.

### Responsibilities

Standardization of workflows and protocols for the appropriate use of ultrasound equipment, data storage requirements, and minimum documentation requirements are essential for the safe and efficient use of POCUS. To meet the needs of the majority of clinical groups that perform POCUS, this includes implementing EBIW to support efficient clinical use of bedside ultrasound imaging. EBIW allows for the seamless integration of ultrasound data into the EHR while minimizing disruption to normal clinical workflows.

Ultrasound device and POCUS management system selection and procurement are critical components of technology governance. The selection of ultrasound devices will be based on technical and interoperability factors within the broader enterprise imaging strategy, taking into account factors such as image quality, ergonomics, data security, authentication, authorization, secure storage and communication, and performance metrics.

### POCUS Manager

POCUS manager systems, often referred to as middleware solutions, serve as the central integration platform within a POCUS program, facilitating workflow management and connectivity between ultrasound devices, imaging archives, and clinical information systems. These solutions play a critical role in enabling efficient clinical workflows while maintaining program quality and compliance.

Understanding the essential functions of a POCUS manager is crucial for program success. Table 1 outlines the core functions that any POCUS manager must provide to support a successful POCUS program. These capabilities represent the minimum requirements for effective program operation and management.

**Table 1:** Core functions of a POCUS manager

Core function	Description
Standard-based integration	Connects with ultrasound devices and medical imaging archives (PACS/VNA) using industry-standard protocols
Vendor-agnostic support	Works with ultrasound devices from multiple manufacturers
EHR integration	Supports encounter-based image workflow (EBIW) integration with the electronic health record (EHR)
Structured documentation	Provides POCUS documentation and reporting capabilities using structured reporting templates
Access management	Implements role-based access management and authentication
Worklist management	Associates patients with their exams through modality worklists
Quality assurance	Enables monitoring and reporting of exam and image quality

Beyond these core functions, POCUS managers may offer additional capabilities that can enhance program effectiveness and support more advanced use cases. Table 2 presents enhanced features of a POCUS manager that organizations should consider based on their specific needs and program maturity.

**Table 2:** Enhanced/desirable features of a POCUS manager

Enhanced feature	Description
Artificial intelligence–augmented reporting	Provides automated assistance in report generation and interpretation
Advanced analytics	Provides comprehensive program management dashboards and metrics
Study organization	Capability to tag and organize studies for clinical, educational, and research purposes
Multi-site management	Provides tools for managing POCUS programs across multiple locations
Teaching integration	Provides support for educational programs and credentialing systems
Mobile support	Allows dedicated mobile applications for image review and quality assurance
Device management	Allows advanced tracking and management of ultrasound device fleet
Research support	Integrates with research databases and analytic tools
Workflow optimization	Provides automated tools for workflow efficiency and program optimization
Custom quality metrics	Configurable quality dashboards and monitoring tools

The workflow integration capabilities of a POCUS manager are particularly crucial for program success. Through integration with the EHR, these systems streamline the process of patient identification and exam documentation. The creation of modality worklists (MWL) ensures accurate patient-exam association and supports efficient clinical workflows. This integration extends to enterprise imaging systems, ensuring proper archival and accessibility of POCUS studies within the broader imaging ecosystem. A POCUS manager should support EBIW by aligning health level 7 (HL7) ORU (Observation Result) messages to pre-built POCUS orders in the EMR so that orders can be created retroactively.

Security considerations are equally important in POCUS manager implementation. Role-based access management ensures appropriate user permissions while maintaining necessary audit trails. The system should support both on-site and remote access needs, particularly for quality assurance activities, while maintaining alignment with organizational security policies. Authentication should integrate seamlessly with existing identity management infrastructure to maintain consistent security standards across the enterprise.

When selecting a POCUS manager solution, organizations should carefully evaluate both core functions and enhanced features against their specific program needs. While all core functions are essential for basic program operation, the selection of enhanced features should align with the organization’s strategic objectives, program maturity, and available resources. Regular assessment of program needs can help identify when additional enhanced features may provide value to the growing program.

### Security

Device and information security are critical components of POCUS technology governance, particularly given the mobile nature of modern ultrasound devices and the need to protect protected health information (PHI). Historically, medical imaging modalities have not implemented robust user authentication, creating significant security risks that must be addressed in modern POCUS implementations.

Effective use authentication and authorization are essential elements of any POCUS program. The POCUS manager solution must support secure user login while maintaining efficient clinical workflows, as documentation often occurs during active patient care. Integration with enterprise authentication mechanisms, such as Single Sign-On (SSO) or Active Directory, helps maintain security standards while providing convenient access for clinicians. Access to the POCUS manager system must be available both on-site and off-site, particularly as programs expand into telehealth and community outreach settings. This accessibility requirement should be carefully considered during system selection and implementation.

The security landscape is further complicated by the variety of POCUS devices in use today. These range from compact cart-based devices to hand-carried laptop-sized units and increasingly include pocket-carried devices that often operate through smartphones or tablets [31]. Each form factor presents unique security challenges that must be addressed. For mobile devices, particularly smartphones and tablets used with handheld ultrasound units, Mobile Device Management (MDM) software provides an essential security layer to protect both devices and patient data.

The trend toward personally owned devices requires specific attention within the security framework. As ultrasound devices become more affordable and portable, individual clinicians increasingly purchase their own equipment. While the decision to allow bring your own device (BYOD) typically lies with the program governance, technology governance must establish clear security protocols for these devices [33]. This includes defining requirements for device integration, implementing appropriate security controls, and ensuring compliance with institutional policies. It is important to note that the use of clinician-owned personal ultrasound devices may impact the billing practices for the medical institution, an aspect covered in detail within the financial governance section.

A comprehensive security approach must also include robust device management and tracking capabilities. Healthcare organizations need to monitor equipment locations, maintain service records, and trace device utilization patterns. This monitoring not only supports security compliance but also provides valuable data for program optimization. Regular analysis of usage patterns helps identify training needs, optimize device deployment, and ensure appropriate maintenance, all of which contribute to the overall security and reliability of the POCUS program.

### Integration

A centralized management and integration approach is necessary when deploying a large, multi-department POCUS program. A centralized approach ensures that the POCUS program is standardized and consistent across all departments and that the documentation and imaging data are seamlessly integrated into the EHR and enterprise medical imaging archives, such as PACS or a VNA system. Leveraging a common platform across all participating POCUS departments has multiple advantages: reduced overall complexity due to the requirement for a single integration method with the EHR and/or VNA, the ability to more easily scale the solution when onboarding new departments or clinical subspecialties, and reduced support/maintenance costs due to the elimination of duplicate or siloed systems that are designed to perform the same overall function.

### Technology Procurement

The technology procurement process for POCUS programs requires a structured governance framework that ensures the strategic acquisition of both devices and supporting systems while maintaining organizational standards and compliance. A dedicated procurement committee, comprising clinical leaders, IT representatives, and procurement specialists, should oversee this process through clearly defined roles and standardized evaluation criteria.

Effective procurement governance begins with establishing clear technical requirements and standards. These include minimum specifications for approved devices, required integration capabilities with existing systems, and security compliance requirements. The governance structure must also address the increasing trend of clinician-owned devices by establishing specific criteria for acceptable personally owned devices and protocols for their integration with institutional systems.

The procurement process should emphasize a thorough technical assessment of potential acquisitions. This includes evaluation of integration capabilities with existing POCUS manager systems, compatibility with current EHR and imaging archive solutions, and verification of support for required workflow patterns and clinical protocols. Vendor management is equally critical, with clearly defined service level agreement requirements and established protocols for ongoing performance monitoring.

Cost management within the procurement framework requires comprehensive planning that goes beyond initial purchase prices. Organizations should develop standardized total cost of ownership (TCO) calculation methods that account for integration costs, IT support requirements, training resources, and infrastructure upgrades, including the need to retire devices that no longer meet the minimum required integration capabilities with existing systems and security compliance requirements. This is particularly important when implementing solutions across multiple departments, where clear budget allocation procedures and cost-sharing models become essential.

Documentation plays a vital role in procurement governance. Organizations should maintain detailed records of procurement decisions, device inventories, deployment locations, and vendor performance history. Standard operating procedures for device acquisition should be clearly documented and regularly updated to reflect changing technological and clinical needs.

The procurement governance framework should remain flexible enough to accommodate technological advances while providing clear guidance for technology acquisition. Regular review and updates of procurement policies ensure continued alignment with organizational goals and regulatory requirements, supporting the long-term sustainability of the POCUS program.

## Information Governance

### Goals

While technology governance is vital to the foundation and success of a POCUS program, information governance is essential to ensure that appropriate data is captured within POCUS workflows. The data has numerous downstream impacts on clinical care, QA, coding, billing, and other domains, such as research and education. Consequently, it is important to understand and plan the mechanisms that will generate and capture data with each occurrence of a POCUS workflow to ensure high-quality and interoperable data. While some POCUS settings may prefer an order-based workflow, an encounter-based imaging workflow (EBIW)^18^ will commonly offer a more efficient workflow for providers. Regardless of the workflow, the necessary data should be captured in a consistent manner.

### Culture and Makeup

It is recommended that a healthcare system establish a multidisciplinary information governance committee to oversee the data collected by their POCUS workflows and how those data elements are defined. The committee should also endeavor to define the specific exam procedures that are allowed to be performed as POCUS and the required content for each exam. Standardization will allow for consistent application of the same exam across different clinical settings and will also allow for a reliable privileging process. It should be noted that appropriate information governance is an essential component of all data captured in healthcare operations, and POCUS information governance should be seen as a subset of an overall enterprise effort at information governance. The committee should include representation from the different clinical specialties that use POCUS as well as health information and security officers (e.g., chief medical information officer, chief information security officer, chief information officer, chief technology officer, or their designees). The committee should be tasked with ensuring that the data collected in POCUS workflows is sufficient to allow for the evaluation of POCUS exams for credentialing, QA processes, and billing of clinical exams as well as supporting education and research endeavors.

Educational exams are a concept unique to POCUS. Educational exams are performed solely for training purposes, have no clinical indication, and are typically not reported to the EHR. Further, trainees commonly require feedback data on the quality of their educational exams as well as their interpretation of the findings. Therefore, these educational exams require thoughtful consideration to ensure that they are performed appropriately and that the relevant information is captured to ensure learners are developing their POCUS skill set appropriately. Based on the American Medical Association policy on privileging for ultrasound imaging (H-230.960), “each hospital medical staff should review and approve criteria for granting ultrasound privileges based upon background and training for the use of ultrasound technology and strongly recommends that these criteria are in accordance with recommended training and education standards developed by each physician's respective specialty” [26]. Although educational exams will be of most interest to academic healthcare systems, it is important to note that these exams can occur in any healthcare system.

### Responsibilities

The information governance committee should consider what information to capture as part of an educational exam, how to identify or label these exams, and where these exams should be stored. One of the most important pieces of information to capture is whether any given exam is an educational exam or a clinically relevant exam. The information must be manually entered by the user at some point in the workflow. The final storage location of educational exams can vary depending on local policies. Similarly, exams performed for research purposes also have their own unique considerations. It is important to think about how these exams will be identified, where the data will be stored, and how exams will be deidentified if necessary.

As the preferred workflow in most clinical departments that use POCUS, EBIW begins with the clinician selecting a patient from the MWL. An HL7 admission, discharge, and transfer (ADT) feed from the EHR commonly provides the necessary patient demographics information [34] to the POCUS manager to produce the MWL. The clinician then enters their own identifier, selects any necessary settings on the modality, and captures images. The captured images are then transmitted to the POCUS manager. The clinician can then use the POCUS manager to document the type of exam performed, enter their findings, and specify whether the exam is clinically relevant (clinical exam) or was obtained for training purposes (educational exam). The use of a POCUS manager for reporting offers the efficiency of allowing the provider to document all necessary information (exam type, educational vs. clinical exam, exam findings, etc.) in a single location with the trade-off being the need to document in a system outside the EHR. In other workflows, such as reporting in the EHR, the need to label the exam as educational or clinical generally needs to occur before the exam is sent to the EHR so the provider will still need to document at least this one piece of information outside the EHR. The images and associated documentation can then be maintained in the POCUS manager and interfaced with different software systems, such as the EHR for textual results and VNA or PACS for archiving images and video clips.

For POCUS practices that utilize an order-based workflow, the first step is order entry, generally in the organization’s enterprise EHR. The order (containing identifiers for the patient, ordering provider, and potentially exam type) is then interfaced, commonly using the HL7 standard [34], to the POCUS manager where it is converted to the DICOM standard for diagnostic imaging and is placed on a MWL. A clinician using the modality to complete a POCUS exam selects the order from the worklist; the remaining steps generally mirror those described above for EBIW.

The data collected during a POCUS workflow can conceptually be split into two categories:

1. Data that is automatically generated by a system
2. Data must be manually entered by a user during the workflow

Generally, automatically generated data is preferred for its reliability; however, it is not possible to automate all data in a POCUS workflow. The members of a POCUS information governance team should first work to determine what data is necessary to capture during a workflow and then work alongside their technical partners to maximize those data that can be automated. The opportunity to automate data capture will vary depending on the workflow, and consequently, it is important that members of the information governance team have at least a rudimentary understanding of data flows in a POCUS infrastructure.

POCUS exam data can be categorized into one of the nine groups (Table 3). Each group may contain one or multiple individual data elements. It is the responsibility of the POCUS information governance team to identify and define the data elements for their organization. For example, one organization may choose to use the National Provider Identifier (NPI) number as their operator identifier, while another organization could opt to use an internal employee ID number or username. Either approach is valid so long as the item is well defined and consistently used within the organization’s POCUS workflows. However, POCUS information governance teams should consider downstream applications of the data when making their selections. For example, the organization that opts to use an NPI number to identify the operator will likely find this beneficial when they begin to consider how to generate a charge for the exam. When considering downstream data uses, organizations should also account for research and educational applications and anticipate the need to remove patient identifiers in some circumstances.

**Table 3:** Point-of-care ultrasound (POCUS) exam data elements

POCUS data group	Type of information flow (automatic or manual)	Comments
Date and time	Automated	Generated from modality
Location	Can be automated	Patient location—department/clinic/facility
Modality identifier	Automated	Technical name: application entity title [AET]
Patient identifiers	Can be automated	Dependent on user adherence to workflow—can be streamlined with the use of a barcode scanner
Operator identifier	Always manual	Can be streamlined with the use of a barcode scanner
Supervisor identifier	Always manual	Optional
Exam type	Can be automated	Workflow dependent
Exam findings	Commonly manual	Includes images, annotations, and findings
Billing codes	Can be automated	Workflow dependent

Manually entered data elements will commonly pose the greatest challenge to reliable and high-quality data collection. POCUS information governance teams should work with their technology governance partners to identify mechanisms to simplify or eliminate manual data processes. In some cases, an operator could identify themselves by selecting a field on the modality screen and either manually enter their ID or scan their badge with a barcode reader. Both require a manual step by the user; however, the second is a simpler process and may lead to greater compliance and fewer errors. One can envision a future state where a modality uses biometrics to identify the operator further simplifying the process of entering the operator identifier during the POCUS workflow.

In summary, the POCUS information governance element recommendations or guidelines are as follows:

1. Establish a multidisciplinary information governance committee tasked with evaluating what information should be captured during POCUS workflows and developing data definitions.
2. Ensure that the information captured during POCUS workflows is sufficient to allow for appropriate clinical care, QA, billing, and where appropriate education and research.
3. Work closely with technology governance teams to simplify POCUS workflow and maximize processes that allow data to be captured automatically when possible.
4. Maintain data policy management with workflows, address regulatory compliance, promote audit readiness, and maintain customer trust.
5. Simplify processes to assist with increasing compliance.

## Financial Governance

### Goals

The financial governance of a POCUS program should account for a multitude of factors that can be framed through appropriate performance, image capture, documentation, and quality improvement related to POCUS examinations. Each of these factors requires allocated resources, which POCUS financial governance needs to anticipate.

### Culture and Makeup

As programs are being designed and implemented, the committees should identify key financial priorities and personnel among the various stakeholders, while considering both monetary and non-monetary return on investment estimates.

### Responsibilities

First, an overarching program strategy should be planned, such as a phased or partial rollout (POCUS implementation initially within a selected area before including other areas over time) or a full healthcare system-wide rollout. A phased rollout may be desired when a healthcare system seeks to understand the costs of program implementation as it allows for discoveries to be made on a local level and to gain information about user acceptance over time. Conversely, a full system-wide rollout may drive POCUS development across the institution, take advantage of one-time endowments, and reduce overall overhead costs. The governance committee should engage stakeholders to determine the best strategy for the healthcare system. Once a strategy is chosen, the committee will need to consider both direct and indirect costs related to equipment (hardware and software), training, clinical and educational oversight, workflow support, and the personnel required compared against the potential return on investment that a POCUS program provides to a healthcare system.

Ultrasound device budgets need to include initial purchase costs and maintenance and other additional costs of the devices, such as those arising from personnel, infrastructure, and software that support their use. In addition, budget projections should include replacement for devices at end-of-life. Decisions on initial device purchases can be challenging. For example, while handheld devices come at a considerably cheaper price point, they typically have a shorter lifespan than cart-based ultrasound devices and may have additional physical and software security concerns and ongoing subscription fees [35]. To properly budget for the program, prior to purchasing devices, it is advisable to involve system IT and technology-management services to vet device security, estimate additional anticipated software and maintenance costs, advise on appropriate procurement processes, and potentially plan for an overall device management strategy. Decisions to purchase warranties on these devices to balance the risk of damaged hardware leading to device loss need to be carefully considered.

Inventory management and security teams may require additional software or hardware, such as Bluetooth or radio frequency identification (RFID) tags to prevent theft and loss. Essential disposable items associated with ultrasound use such as gel, transducer covers, echogenic needles, and electrocardiogram leads also need to be included in the budget, in addition to expenses associated with cleaning and disinfection protocols, like disinfectant wipes or high-level disinfection. Remote or low-resource healthcare systems may need to consider costs for procurement (taxes, transportation, etc.), power, cellular and/or WiFi network connection, and image storage mechanisms.

With regards to the user, POCUS programs need to budget for adequate education, training, and oversight of clinician-users. Expenses may include ultrasound simulators, task trainers (e.g., phantoms), other devices or software that support learning as well as workshop overhead (which can include devices, educational space, and standardized patients), paid faculty time for providing training and quality assurance of both educational and clinical images, and time that learners/trainers are out of clinical space or utilizing learning resources. Programs may choose to monitor specific POCUS-related metrics or support research activities, and these activities may need to be worked into the budget. Quality assurance, credentialing, and privileging processes can be time-intensive and likely will require clinician oversight. Specific requirements are often made at a local healthcare system level; therefore, discussion with engaged stakeholders is important to determine the time needed to be allocated for these activities based on the set expectations.

As POCUS workflows are enhanced by either leveraging the organization’s enterprise imaging platform or through a dedicated POCUS manager, costs for enterprise imaging implementation, server or cloud-based architectures, vendor support and maintenance, and internal expenses for resources such as IT staff must be considered. Plans for policy-based image retention of these images may be helpful to alleviate resources that are associated with storage requirements. Lastly, coding and billing department activities need to be weighed in the financial evaluation, and automation of billing processes should be considered, which can be done with either order-based workflow or EBIW.

POCUS program expenses can be offset by a return on investment from the capturing of services rendered and indirectly through improved quality indicators and other patient-related outcomes. In the USA, POCUS charges are typically separated into professional and technical components. In ambulatory settings, POCUS activities usually recoup both these charges (global charge) in clinically indicated studies, while technical charges in the inpatient setting are often bundled into diagnosis-related groups, and thus, reimbursement that the healthcare system directly receives from inpatient POCUS activities is significantly limited [31, 36]. Technical charges may only be applied by the healthcare system if it owns the ultrasound devices. As handheld ultrasounds become more affordable for individual purchase, clinician-owned devices are prevalent, and technical charges cannot be applied for POCUS examinations performed on these devices. Additionally, many facilities may choose to utilize demonstration machines while exploring purchasing options or for other reasons. In this case, these cannot be used for clinical work and, in general, cannot generate charges from captured studies. Creating a plan for system purchase is important if the system wishes to recoup these technical charges. Furthermore, insurances determine reimbursement for POCUS activities, and there are typically significant differences between charges applied and charges captured. Regional factors like reimbursement, supplies costs, and specific healthcare regulations need to be accounted for. For an example estimation of return on investment, please see Table 4.

**Table 4:**
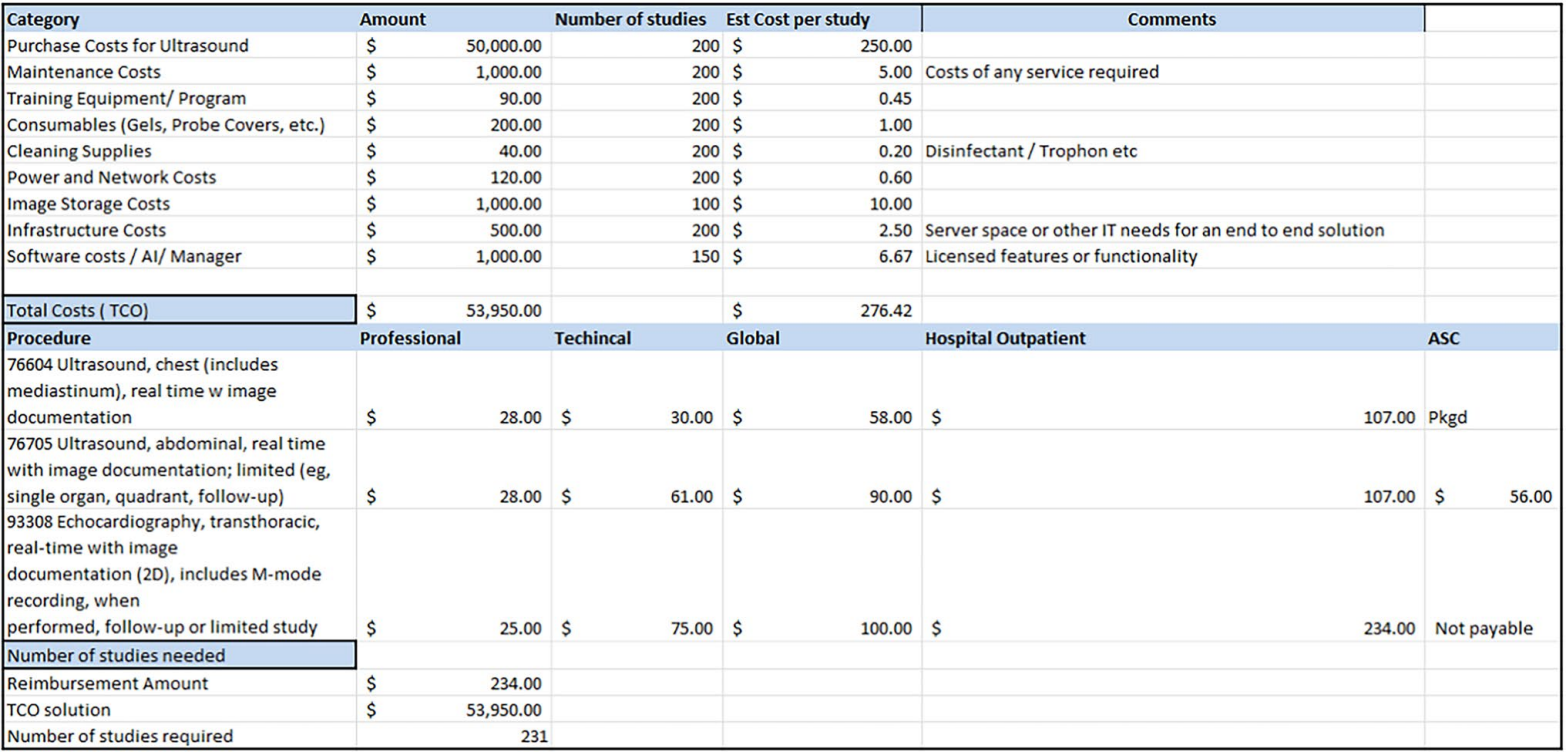
Example of a POCUS Total Cost of Ownership (TCO) and potential return on investment (ROI) estimation spreadsheet

Legend: The table is an example of a TCO and potential ROI spreadsheet. These tools can help committees assess the direct and indirect costs of a POCUS Program. This table is meant to be a reference and may not reflect the true costs of inputs or reimbursement for outputs. It is recommended that the user develop their own spreadsheet using their own values to populate the table. Reimbursement from https://www.cms.gov

The importance of careful consideration of multiple cost–benefit-related factors by the financial governance committee cannot be understated (Table 5). While there is growing support that POCUS-informed care leads to improved patient safety–related outcomes such as improved diagnosis, time to treatment, improved procedural safety, and prediction of prognosis [37], how much cost-savings a system will incur due to POCUS remains poorly understood and needs to be explored on system-wide levels [31]. Financial risks to a system as a result of lack of training and oversight, inappropriate use of POCUS, or improper image storage may serve as a powerful disincentive for institutional buy-in. Additionally, a robust POCUS program may aid in the recruitment and retention of clinical staff. On a global level, we are facing increasing impacts of climate change. Ultrasound remains the diagnostic imaging modality of choice given it has the lowest carbon footprint [38]. Finally, more research is needed to understand how the cost of performing POCUS impacts patient-experienced costs.

**Table 5:** Costs and benefits of establishing and maintaining a point-of-care (POCUS) Program

**Costs**
Ultrasound devices (purchase and maintenance costs)
Associated ultrasound equipment (e.g., cleaning supplies, gel)
Software costs (POCUS manager, security)
Power and network costs
Personnel time (training, IT, administration, quality assurance, etc.)
Program assessment tools
Research program infrastructure and time
Image storage
Ultrasound training equipment and resources
**Benefits**
POCUS charges captured
Improved patient safety outcomes
Patient efficiency measures
Academic output
Clinician satisfaction and wellbeing
Clinician recruitment and retention
Environmental impact (lower carbon emissions compared to other imaging modalities)
Improved accuracy in obtaining images

## Conclusion

POCUS has advanced healthcare by providing rapid diagnostic information at the bedside. Deploying POCUS with the appropriate governance in place reinforces the growth and success of a healthcare system’s program. As healthcare systems increasingly adopt POCUS technology, building a robust governance structure consisting of program, clinical, technology, information, and financial governance is essential for ensuring the program’s effectiveness, success, and sustainability. By addressing these essential governance components, along with the establishment of institutional policies, healthcare organizations can optimize their use of POCUS, streamline operational efficiency, and improve patient care and satisfaction.
